# Sensory acceptability of biofortified foods and food products: a systematic review

**DOI:** 10.1093/nutrit/nuad100

**Published:** 2023-08-27

**Authors:** Samantha L Huey, Arini Bhargava, Valerie M Friesen, Elsa M Konieczynski, Jesse T Krisher, Mduduzi N N Mbuya, Neel H Mehta, Eva Monterrosa, Annette M Nyangaresi, Saurabh Mehta

**Affiliations:** Division of Nutritional Sciences, Cornell University, Ithaca, New York, USA; Program in International Nutrition, Cornell University, Ithaca, New York, USA; Center for Precision Nutrition and Health, Cornell University, Ithaca, New York, USA; Division of Nutritional Sciences, Cornell University, Ithaca, New York, USA; Global Alliance for Improved Nutrition, Geneva, Switzerland; Division of Nutritional Sciences, Cornell University, Ithaca, New York, USA; Division of Nutritional Sciences, Cornell University, Ithaca, New York, USA; Global Alliance for Improved Nutrition, Washington, DC, USA; Division of Nutritional Sciences, Cornell University, Ithaca, New York, USA; Global Alliance for Improved Nutrition, Geneva, Switzerland; Global Alliance for Improved Nutrition, Nairobi, Kenya; Division of Nutritional Sciences, Cornell University, Ithaca, New York, USA; Program in International Nutrition, Cornell University, Ithaca, New York, USA; Center for Precision Nutrition and Health, Cornell University, Ithaca, New York, USA

**Keywords:** acceptability, biofortification, iron, vitamin A, zinc

## Abstract

**Context:**

It is important to understand the sensory acceptability of biofortified food products among target population groups if biofortification is to be realized as a sustainable strategy for mitigation of micronutrient deficiencies, able to be scaled up and applied through programs.

**Objective:**

This systemic review aims to summarize and synthesize the sensory acceptability of conventionally bred iron-, zinc-, and provitamin A–biofortified food products.

**Data Sources:**

MEDLINE (PubMed), AGRICOLA, AgEcon, CABI Abstracts (Web of Science), and organizational websites (eg, those of HarvestPlus and CGIAR and their partners) were searched for relevant articles. No access to any market research that may have been internally conducted for the commercial biofortified food products was available.

**Data Extraction:**

This review identified articles measuring the sensory acceptability of conventionally bred biofortified food products. Extraction of the hedonic ratings of food products was performed.

**Data Analysis:**

An “Acceptability Index %” was defined based on hedonic scoring to determine an overall rating, and used to categorize biofortified food products as “acceptable” (≥70%) or “not acceptable” (<70%). Additionally, this review narratively synthesized studies using methods other than hedonic scoring for assessing sensory acceptability.

**Conclusions:**

Forty-nine studies assessed the acceptability of 10 biofortified crops among children and adults, in mostly rural, low-income settings across Africa, Latin America, and India; food products made from mineral and provitamin A–biofortified food products were generally acceptable. Compared with studies on provitamin–A biofortified food products, few studies (1 to 2 each) on mineral-enhanced crops such as rice, cowpeas, lentils, and wheat were found, limiting the generalizability of the findings. Similarly, few studies examined stored biofortified food products. Few commercial food products have so far been developed, although new varieties of crops are being continuously tested and released globally. Certain crop varieties were found to be acceptable while others were not, suggesting that particular varieties should be prioritized for scale-up. Determining sensory acceptability of biofortified food products is important for informing programmatic scale-up and implementation across diverse populations and settings.

## INTRODUCTION

Biofortification, ie, the process of increasing the concentrations and bioavailability of essential nutrients in staple crops by conventional plant breeding, agronomic techniques, and genetic engineering, is a promising approach to combating micronutrient deficiencies in at-risk populations around the world, estimated to affect 1 in 3 women of reproductive age and 1 in 2 pre-school–aged children globally.^1^ Ultimately, biofortification has the potential to serve as an economically and environmentally sustainable means of contributing to addressing the burden of micronutrient deficiencies at a population level via already existing food systems.

To be fully realized as a sustainable solution for mitigating micronutrient deficiencies, it is important to understand how well the sensory characteristics of biofortified foods and food products are accepted among target population groups, particularly in comparison to substitute foods made from nonbiofortified conventional crop varieties. Staple crops commonly targeted for biofortification are often traditionally consumed in the diets of many populations and can include sweet potato, pearl millet, wheat, lentils, cassava, rice, beans, and maize.^2^ However, the process of biofortifying foods may lead to visible or sensory changes in the conventional crop varieties and the resulting foods (eg, crops with greater provitamin A content are likely to be yellow or orange in color compared with their conventionally white counterparts, and biofortified orange sweet potato (OSP) is likely to be mushy or soft compared with white sweet potato^3^). Previous reviews have summarized the acceptability of biofortified foods in low- and middle-income countries.^3^^,^^4^ These reviews found that, broadly, biofortified foods were acceptable among consumers. However, the findings were limited by the lack of biofortified food dissemination and availability in the populations being studied and therefore partially relied on “hypothetical acceptability”, ie, based on interviews to ascertain the customer’s own perceived likelihood of accepting a biofortified food after hearing a description of that food, rather than based on directly comparing the acceptability of biofortified foods and nonbiofortified foods through empirical evaluation (hedonic testing, eg, actual tasting and consumption). Further, these reviews did not always record which biofortified food varieties were examined, though it was noted that the variety tested was sometimes an important factor in determining the acceptability of the sensory characteristics.^3^ Understanding the sensory acceptance of biofortified foods among target populations, ascertained through more direct methods, comparing both the sensory acceptance of nonbiofortified foods with that of biofortified foods, and comparing that of biofortified crop varieties, is important for informing food product development and potentially which varieties to emphasize for growing and commercialization. As a note, examining the acceptability of adopting biofortified crops by farmers, and their uptake, is beyond the scope of this review and will be covered separately.^5^

The objective of this review was to determine and summarize the sensory acceptability of food and food products made from conventionally bred iron-, zinc-, and provitamin A–biofortified staple crops, measured using hedonic scoring, or other methods, in which the biofortified food product was directly tested and compared against the same foods made with (a) other varieties of that biofortified crop or (b) nonbiofortified foods.

## METHODS

The protocol for this review was registered on PROSPERO (ID: 254461, no. CRD42021254461), the international prospective register of systematic reviews of the University of York and the National Institute for Health Research, on June 11, 2021.^5^

### Inclusion and exclusion criteria

Our eligibility criteria are summarized in the PICOS (participants, interventions, comparisons, outcomes, and study design) format in Table 1.

**Table 1:** PICOS criteria for inclusion of studies

Parameter	Criterion
Population	Any human population, including infants, children, and adults
Intervention	Conventionally bred biofortified crops-based food products
Comparator	Control crops, either (A) a nonbiofortified (ie, control) version of the same food or food product made using nonbiofortified crops; or (B) food products industrially fortified with the same micronutrient
Outcome	Sensory acceptability, as assessed using hedonic scoring (including facial hedonic scoring) and other scales such as: Just About Right (JAR); children’s food intake (amount of food consumed); Quantitative Descriptive Analysis (QDA); paired preference test
Study design	We included studies wherein biofortified crop-based foods were tested empirically by consumers, panelists, and households, and did not include results based on modeling or hypothesized judgements.

### Participants

Any human population was considered eligible for inclusion, including populations of infants, children, and adults.

### Interventions

Included studies utilized biofortified crops–based foods and food products, including those that have undergone processing post-harvest, that have been delivered in the form of food products (as defined by trialists). Crops included those biofortified by conventional plant breeding approaches. Interventions utilizing agronomic biofortification methods, genetic engineering–based biofortification methods, or animal-based biofortified foods (such as dairy products or meat from animals that consumed biofortified feed) were excluded from this review. Additionally, protein-biofortified crops such as quality protein maize (QPM) were excluded, allowing a focus on micronutrient biofortification.

### Comparators

Comparators included either (a) a nonbiofortified [ie, control] version of the same food or a food product made using nonbiofortified crops; or (b) food products industrially fortified with the same micronutrient.

### Primary outcomes

1. Sensory acceptability: sensory parameters (eg, taste/flavor, smell/odor, appearance/color, texture/mouthfeel, overall, or as defined by trialists), as assessed using a hedonic scale including the facial hedonic scale.

### Secondary outcomes

2. Other scales:
  - Just About Right (JAR)
  - Children’s food intake (amount of food consumed)
  - Quantitative Descriptive Analysis (QDA) method
  - Paired preference test

### Study designs

This review included studies wherein biofortified crop–based foods were tested empirically by consumers, panelists, or households, and did not include studies in which the results were derived from modeling or hypothesized judgments. For example, the analysis included only sensory acceptability studies in which the food or food product was directly tasted and/or consumed by participants to inform their hedonic scale ratings.

### Methods for evaluating acceptability

#### Sensory acceptability

This section briefly describes the methods for analyzing the sensory acceptability of food products, including hedonic testing and variants thereof, ie, JAR analysis, grams eaten, and paired preference tests. The reader is referred to a previous review for a detailed description of sensory evaluation methods and hedonic testing methods.^4^

#### Hedonic scale

Hedonic testing measures the degree to which a consumer likes, accepts, or prefers a given product.^4^ Scales can range between 1 and 5, 7, 9, or higher, with a 9-point scale being the most common. In a 9-point hedonic scale, there is a midpoint of 5 (considered neutral or neither like nor dislike), with 4 positive and 4 negative categories or verbal anchors per side, ranging from “dislike extremely” to “like extremely.”^6^ After participants test the food product, they indicate which number represents their opinion. This simplicity means the testing is accessible to a large population and does not require extensive participant training before use. Additionally, this method can be used for testing overall acceptability in addition to more specific sensory characteristics (eg, smell, texture). In 1 study, among children over 5 years old, 9-point scales discriminated better than 7-point scales.^7^ However, due to the small number of sensory parameter categories (usually 4 or 5) and end-point avoidance, ceiling effects may occur.^8^^,^^9^ This method is most reliable with sample sizes of n ≥ 60 participants, so it is not appropriate for smaller-scale studies.^10^ Additionally, for children, it may be more useful to use a verbal liking scale and using the terminologies of Peryam & Kroll (P&K) – instead of “like extremely” and “dislike extremely”, the terms “super good” and “super bad” are used, respectively.^7^ Additional details, advantages, and limitations of hedonic scale testing are discussed in a previous review.^8^

#### Facial hedonic scale

A facial hedonic scale, or modified category scale, may be used instead of the traditional hedonic scale when surveying populations in which illiteracy is prevalent or among children. The scale includes text and pictures of faces with various expressions (emoticons) to show the range in acceptability from “dislike very much” to “like very much”. After tasting the food product, participants select the emoticon that represents their liking of the sample. The range of this scale varies from study to study, but the most common range is 5 points. In cases where specific sensory attributes are being measured (eg, smell, texture) in lower-literacy populations, it is important that researchers educate the participants on the attribute before testing. Additionally, it may be helpful to support results from the facial hedonic scale with the use of another method, such as a paired preference test, which is suitable for semiliterate and illiterate populations.^11^

#### Just-About-Right sensory analysis

The JAR scale is a bipolar measurement using 2 semantically opposite anchors at either end of the scale.^12^ The center point is called “Just About Right” or “Just Right” and assumed to be a participant’s ideal level, and the product may be evaluated as deviation from this ideal level, for example, “Too Little” or “Too Much”.^12^ The JAR scale typically targets more specific sensory characteristics than a traditional hedonic scale (eg, fermented odor, crumbliness). A weakness of this method is that it requires 3 decisions on behalf of the consumer: (1) perception of the intensity of an attribute; (2) the location of the consumer’s optimal point; and (3) comparing the difference between perceived intensity and this ideal point.^12^

#### Grams eaten (child acceptability)

Children who are beginning to consume solid foods may be too young to participate in traditional sensory acceptability assessment methods.^13–15^ In this method, children are given a weighted sample (additional servings are available ad libitum) and are fed until the child refuses food. The remaining food is weighted to calculate the amount consumed. A greater quantity of food consumed correlates to a higher acceptability and vice versa. Feeding studies have found that young children’s acceptability by food intake may be more reliable than adult’s measurements, which may be biased.^13^ With this method, it is important that the mothers and children are randomized separately, and that the mothers eat after their children to avoid influencing the child’s acceptability.

#### Quantitative Descriptive Analysis

QDA is a method based on the understanding that humans are better at perceiving relative sensory differences than they are at perceiving absolute differences.^9^ Unlike other methods discussed, QDA requires a smaller panel of 10–12 trained individuals. A line scale of about 15.24 cm goes from left to right in increasing intensities (eg, weak to strong).^9^ Panelists score food products using the scale, ensuring a relative, rather than absolute, measurement.^11^ The results of this method are traditionally presented graphically in a “spider web.”^9^

#### Paired preference test

Young children’s acceptability of food products cannot be measured using standard methods due to illiteracy and reduced comprehension. However, a paired preference test has been found to be reliable when testing children older than 2 years.^16^ In this test, children taste a sample of a control food and a sample of the modified food. They then indicate which sample was preferred. This method is useful for including very young children and semi-literate/illiterate populations in studies, but its simplicity also means that the degree of acceptability cannot be measured. Thus, comparisons between food products are less reliably made.

### Literature search

A search of relevant literature databases was conducted to include: MEDLINE (PubMed), AGRICOLA, AgEcon, and CABI Abstracts (Web of Science), and organizational websites (eg, Harvest Plus, CGIAR, and partners). As a preliminary assessment of the literature on biofortification, a broad search was conducted in MEDLINE (PubMed) on March 29, 2021, using the following key terms: “Biofortification”[MeSH] OR “biofortif*”[tiab] OR “bio-fortif*”[tiab]. This resulted in 1434 results. After screening these results and ascertaining key words to use for increasing the sensitivity of the search, the team conducted searches in additional databases, using broader or narrower searching depending on the topic focus of the database. These searches, including the original MEDLINE search, are summarized in Table 2. Organization websites were also hand-searched (Table 3). Additionally, 1147 potential citations outside of the original search were identified during the screening process. These included studies that were: cited in review papers but did not include variations of the term “biofortification” in their abstracts; not indexed in any of the literature databases described above and were thus missed by the original search; published after March 29, 2021, identified from the table of contents alert feeds of journals. Some of the latter included full-text versions of conference abstracts that were found and included in the original screening pool.

**Table 2:** Search strategy across included databases

Database name	Final search string	Date of search	Records
MEDLINE	Biofortification[MeSH] OR biofortif*[tiab] OR “bio-fortif*“[tiab]	2021-03-09	1434
AgEcon	All of the words [biofortif*] in All Fields OR All of the words [bio-fortif*] in All fields	2021-04-07	73
AGRICOLA	TX (biofortif* OR bio-fortif*) AND TX (Adopt* OR Farmer* OR Household* OR Accept* OR Sensory OR DALY OR “disability adjusted life year*” OR Market* OR School meal program* OR Retention OR Mill* OR Process* OR Stor* OR Cook* OR Polish* OR Bioavailab* OR Cost-effectiveness OR Bioaccessib* OR Bioactiv* OR Efficacy)	2021-04-07	722
CAB Abstracts	TS=biofortif* OR TS=bio-fortif* AND TS=(Adopt* OR Farmer* OR Household* OR Accept* OR Sensory OR DALY OR “disability adjusted life year*” OR Market* OR School meal program* OR Retention OR Mill* OR Process* OR Stor* OR Cook* OR Polish* OR Bioavailab* OR Cost-effectiveness OR Bioaccessib* OR Bioactiv* OR Efficacy)	2021-04-07	1538

**Table 3:** Results from hand-searching organization websites

Organization website	Studies identified on April 7, 2021, and added to screening pool
HarvestPlus	75 (manual)
CIMMYT Publications Repository	0 (captured in other databases)
IITA	2 (manual)
CIAT	0 (captured in other databases)
IRRI	0 (captured in other databases)
ICRISAT	151=”biofortif*”
ICARDA	0 (irrelevant)
**TOTAL**	**228**

### Data screening and extraction

S.L.H., N.H.M., E.M.K., and A.B. independently screened all records for eligibility, first at the title/abstract level and subsequently at the full-text screening level. Each record was screened by 2 review authors. Eligible articles were those that indicated they examined biofortified food products for sensory acceptability, including through hedonic testing and other methods such as JAR, QDA, etc.

S.L.H., J.T.K., N.H.M., E.M.K., and A.B. used a subset of articles to improve consistency among the review authors. Consistency was improved in adding additional data extraction fields such as the number of points on a hedonic scale, which varied by study. The review considered all publications, trial registrations, and meeting abstracts reporting on the same population/study as 1 study unit, and cited these as such. This reviewprioritized analyzing the data reported in peer-reviewed published articles but also cite the data published as meeting abstracts or conference proceedings.

S.L.H., N.H.M., E.M.K., and A.B. extracted data fro, each identified study, including: study level details, including authors or research group, study year, funding sources, and location; method details, including study design, population, and intervention characteristics; outcomes of interest, including hedonic scores (mean or median with variance in standard deviation [SD] or interquartile range [IQR]) and other acceptability-related outcomes such as JAR, QDA, etc. Missing data was not imputed. Plot Digitizer software was used to extract raw data from figures as appropriate (https://plotdigitizer.sourceforge.net/).

### Data synthesis and analysis

For each study that used the hedonic scale or facial hedonic scale method, the mean (SD) or median (IQR) hedonic rating for each parameter (eg, taste, aroma, etc.) for each biofortified food was recorded. The mean rank score was converted to a percentage of the sum of the number of hedonic scale ranks to standardize this outcome across studies with different scale lengths; for example, a mean score of 4.00 out of a 5-point hedonic scale, or a 7.20 out of a 9-point hedonic scale, would both convert to 80% acceptable. This percentage is known as the “acceptance index,” as described previously.^17^

A cutoff of ≥70% was used to classify each hedonic scale parameter as having good sensory acceptance, according to previous work.^17^^,^^18^ This review considered a product to have good sensory acceptance overall if at least half of the sensory parameters had an acceptance index of ≥70%.

If biofortified foods are acceptable compared with nonbiofortified foods, or if both types of foods result in ≥70% acceptability indices, then biofortified foods were considered to be adequately acceptable.^14^^,^^15^

For studies that used the hedonic scale but reported data as the number of participants who chose each level of the scale, instead of reporting a mean value, the mean was back-calculated manually by weighting the number of participants by the hedonic value and dividing by the total number of participants.

In general, hedonic scales show the minimum rank (eg, 1) as being the least acceptable, and the maximum rank (on a 5-point scale, a rank of 5) as being most acceptable. Any hedonic scales that used opposite scoring (ie, 1 as most acceptable, 5 as least acceptable) were converted to maintain consistency in our review.

Studies that used sensory acceptability assessment methods other than a hedonic scale were synthesized narratively.

## RESULTS

**Figure 1:**
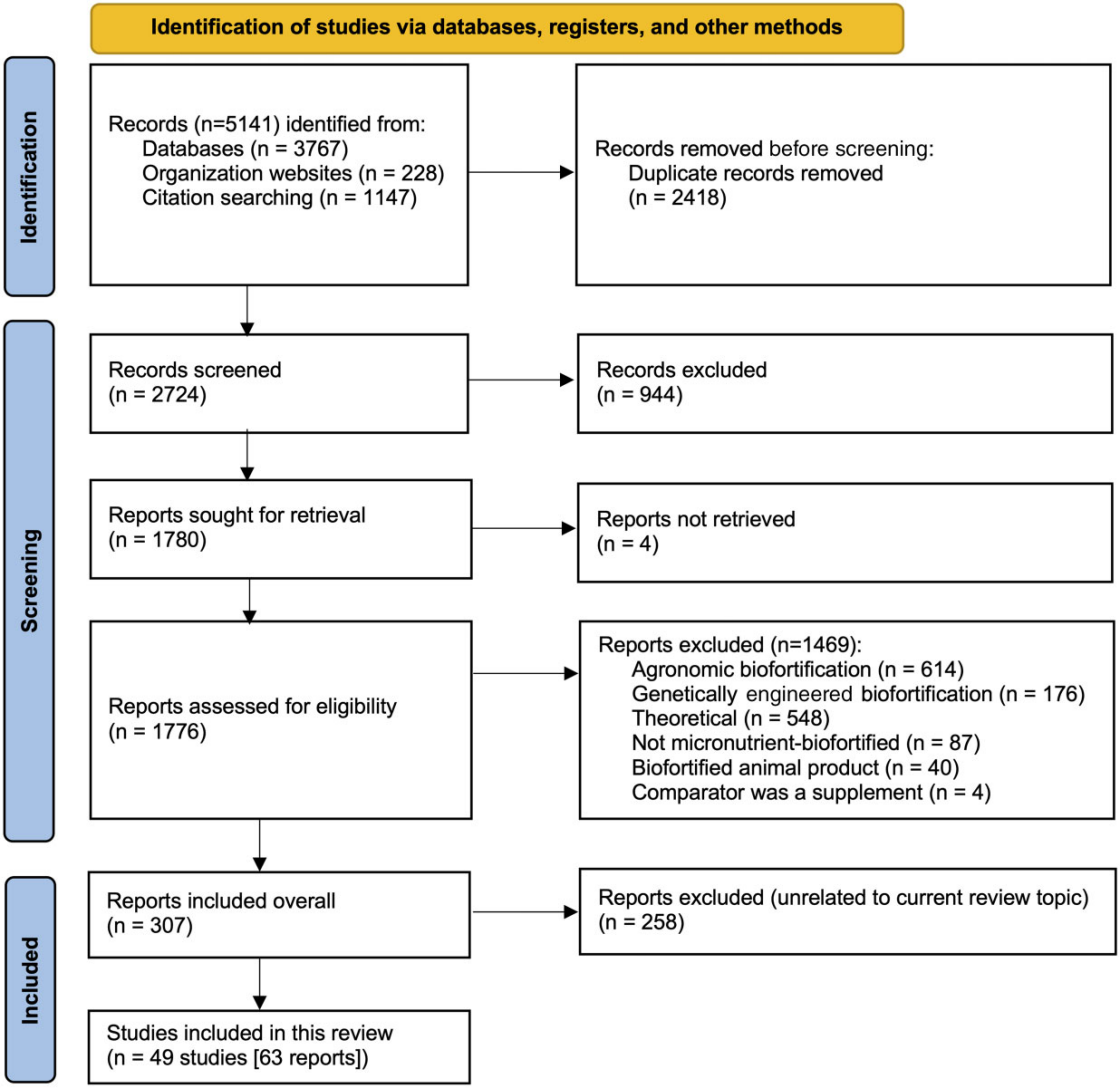
**Flowchart of search and selection process.** ^82^

For the 4 review topics, a total of 5141 records (Figure 1) were identified. Overall, 305 eligible records were found across the 4 review topics outlined previously. Excluded studies did not report on sensory acceptability. For this review topic, 49 studies (63 reports) on acceptability across 10 types of crops were included: 14 studies on OSP, 13 studies on maize, 6 studies on beans, 8 studies on cassava, 5 studies on pearl millet, 2 studies on rice, 1 study on cowpeas, and 1 study that examined both separately and in combination OSP, pearl millet, lentils, and wheat. Nine studies were done in South Africa, 9 in Nigeria, 6 each in India and Brazil, 4 in Colombia, 2 each in Rwanda, Uganda, and sub-Saharan Africa, and 1 each in Bolivia, Burkina Faso, Ghana, Guatemala, Malawi, Mozambique, Panama, Tanzania, Kenya, and Zambia. All controls used in these studies were nonbiofortified versions of the same crop; no studies examined industrially fortified crops as comparators.

### Summary of overall results

The most frequently used hedonic scale in this review was the 9-point hedonic scale, although 4-point, 5-point, and 7-point hedonic scales were also used (Tables  4–10, see Tables S1–S10 in the Supporting Information online). All scales are represented by “dislike extremely” at the extreme minimum (1) and “like extremely” at the maximum (either as originally constructed or as converted by us). Several studies used methods other than hedonic scale testing for assessing sensory acceptability, such as JAR analysis, weighed intakes, QDA, and a paired preference test.

**Table 4:** Summary of studies investigating sensory acceptability of provitamin-A biofortified orange sweet potato (OSP) via hedonic score testing

Population, N	Hedonic scale	Varieties investigated	Food product	Acceptability result^a^ for biofortified food product	Reference
Adults in rural Rwanda, N = 1073	5-point	NR	OSP juice 80% OSP + 20% pineapple juice	Acceptable without pineapple juice added, with nutritional information given	Bocher et al (2019)^22^
Women in Panama, N = 50	5-point, facial	NR	Roasted Tamale Pesada Soda	Acceptable	Britton et al (2017)^23^
Adults in Uganda, N = 40	9-point	Ejumula SPK004/1/1 Tanzania variety Nakakande (control^b^)	Boiled	Sensory hedonic scores not reported	Chowdhury et al (2009, (2011)^20^^,^^21^
Breastfeeding mothers in rural India, N = 52	9-point	Kamalasundari Local variety (control)	Chutney Rasam Thick Shake Barfi Payasam Sharbat	Acceptable	Gannon et al (2019)^24^
Adults in rural South Africa, N = 120	5-point, facial	A40 A45 (control)	Boiled	Acceptable	Govender et al (2019)^25^
Untrained university students and staff in Kenya, N = 25	7-point	Kemb 10 Dutch Robyjin (control)	Chips (crisps) (prepared using either corn or palm oil)	Acceptable	Hagenimana et al (1998)^26^
Adults and children in rural Malawi, N = 210	5-point	Chipika Kadyaubwerere Zondeni Kenya (control)	Boiled	Chipika not acceptable Zondeni acceptable Kadyaubwerere generally acceptable	Hummel et al (2018)^27^
Adults in Brazil, N = 100	9-point	Beauregard	Dry enriched cookie (50% of dry sorghum flour + 50% of sweet potato flour) Extruded enriched cookie (50% of extruded sorghum flour + 50% of sweet potato flour)	Acceptable	Infante et al (2017)^28^
Adults and children in South Africa, N = 930	5 = point, facial	Resisto	Chips Doughnuts Juice Veggies	Acceptable	Laurie (2012)^29^
University students and staff in India, N = 100	4-point	S-61 S-594 S-1156 S-1281 SV-98 362-7 IGSP-15 CIPSWA-2 187017-1 440038 440127 420027 ST-14 Kamala Sundari 90/101	Boiled	Acceptable except for 187017-1, 420027	Mitra et al (2010)^30^
Teachers, school employees, students in Brazil, N = 32	9-point	Beauregard	Bread (20% OSP, 80% conventional wheat) Bread (40% OSP, 60% conventional wheat) Bread (60% OSP, 40% conventional wheat)	Acceptable	Nunes et al (2016)^31^
Semi-trained panelists in rural Nigeria, N = 20	9-point	NR	Boiled with or without skin for 10 or 15 minutes	Acceptable except for boiled without skin for 15 minutes	Pessu et al (2020)^32^
Children 8 y–10 y old, in urban Brazil, N = 100	5-point	Beauregard	Cake	Acceptable	Ramos et al. (2019)^17^
Untrained women and men in Brazil, N = 104	9-point	Beauregard	Cake (OSP: 35.86, 40, 50, 64.14, or 60 g/100 g; sunflower oil: 1.3, 3, 7, 11, or 12.65 g/100 g)	Acceptable	Silva et al (2019)^33^
Preschool and school children (N = 94), mothers (N = 59) in rural Tanzania	7-point, facial	Karote DSM Resisto Polista (control) Sinia B (control)	Boiled	Acceptable	Tomlins et al (2007)^19^

a Foods were considered acceptable if they had an overall sensory acceptability score of ≥70%.

b Control refers to a nonbiofortified, non-industrially fortified, conventional crop.

*Abbreviations:* NR, not reported; OSP, orange sweet potato.

**Table 5:** Summary of studies investigating sensory acceptability of provitamin A-biofortified maize via hedonic score testing

Population, N	Hedonic scale	Varieties investigated	Food product	Acceptability result^a^ for biofortified food product	Reference
Nursing mothers in Nigeria, N = 10	4-point	A0905-32 Common yellow maize (control^b^)	Porridge (40%–50% fermented maize flower, 20% malted maize flour, 25%–30% soybean flour, ± 10% sugar, ± 5% crayfish powder)	Acceptable	Alabi (2021)^39^
Trained panelists in rural subSaharan Africa, N = 10	9-point	ACR97TZL-CCOMP1-Y-S3-13-1-B-B-B-B-B-B-B/9450xKI 21-3-2-2-1-3-B-B-B-B-B-B-B-B-B (GT-MAS: Gk x BABANGOYO x GT-MAS: Gk)-2-1-3-1-B-B-B-B-B-B-B-B-B-B/(MP420 x 4001 x MP420)-3-1-2-1-B-B-B- (KU1409/KU1414-SR/KVI43)-S2-4-1- BB/4001xB73LPAx4001-33-2-1-B*4 (KU1409/KU1414-SR/NC298)-S2-8-1-BB/9450xKI21-1-5-3 2-2-B*5 (KU1409/KU1414-SR/NC298)-S2-7-1-BB/9450xKI21-7-3-1-2-4-B*4 (KU1409/KU1414-SR/KUI2007)-S2-3-2-BB/9450xKI21-1-5-2-1-2-B*5 9450xKI21-7-2-1-2-B*4/KU1409xMO17LPAxKU1409-27-3-1-1-B*7 Oba Super-II	Boiled after 20 d, 27 d, or 34 d of pollination	Acceptable in maize after 20 d of pollination, not acceptable for the majority of varietals at 27 d and 34 d of pollination	Alamu et al (2014)^40^
Adolescents and adults in rural South Africa, N = 59	5-point, facial	PVA pool A PVA pool H White hybrid (control)	Phutu	Acceptable	Amod et al (2016)^41^
Adult consumers (farmers) in rural sub-Saharan Africa, N = 54	9-point	PVAH-62 White maize (control)	Amahewu with wheat bran inoculum or malted maize inoculum, ± starter culture	Not acceptable	Awobusuyi et al (2016)^42^
Adult consumers in South Africa, N = 70	9-point	PVAH-62 White maize (control)	Amahewu with roasted bambara flour	Acceptable	Awobusuyi and Siwela, (2019)^43^
Adult consumers in Ghana, N = 703	5-point	Orange maize Yellow maize White maize (control)	Kenkey	Acceptable	Banerji et al (2015, 2018); De Groote et al (2010)^44–46^
Adult consumers in rural South Africa, N = 60	5-point	PVAH-27-49 PVAH-1-26 PVAH-50-75 PVAH-79-100 White maize (control)	Stiff porridge	Acceptable	Beswa et al (2020)^47^
Adults in rural South Africa, N = 50	5-point	PVA PVAH-27-49 PVAH-1-26 PVAH-50-75 PVAH-79-100	Maize snack with 0%–3% amaranth	Not acceptable	Beswa et al (2016)^48^
Female caregivers in rural South Africa, N = 60	5-point	Deep orange maize Medium orange maize White maize (control)	Porridge	Acceptable	Govender et al (2014)^49^
Adults in rural South Africa, N = 120	5-point, facial	White maize (WE-3172) (control) PVA A	Phutu and chicken curry Phutu and cabbage curry Phutu and Bambara groundnut curry	Acceptable	Govender et al (2019)^25^
Adults in rural Zambia, N = 478	5-point	Prototype high provitamin A orange maize Yellow maize (control) White maize (control)	Nshima	Acceptable	Meenakshi et al (2012)^50^
Secondary school children (N = 54) and adults (N = 50) in rural South Africa	5-point, facial	KP-78 KP-79 KP-77 Commercial white maize (SC-701) (control)	Phutu Thin porridge Samp	Not acceptable	Pillay et al (2011)^51^
Adolescents and adults (14 y–70 y) in urban Mozambique, N = 201	5-point	Orange maize Isogenic white maize (control) White maize (control)	Nshima^c^	Not acceptable	Stevens and Winter-Nelson, (2008)^38^

a Foods were considered acceptable if they had an overall sensory acceptability score of ≥70%.

b Control refers to a nonbiofortified, non-industrially fortified, conventional crop.

c In this study the food product was referenced as “Xhima.”

**Table 6:** Summary of studies investigating sensory acceptability of iron-biofortified beans via hedonic score testing

Population, N	Hedonic scale	Varieties investigated	Food product	Acceptability result^a^ for biofortified food product	Reference
School children in rural or urban Colombia, N = 348	5-point, facial	BIO-101 BIO-107 Local varieties (control) Local (Diacol-Calima) (control^b^)	Cooked, with vegetable sauce	Acceptable	Beintema et al (2018)^53^
Children (7-11 years)in rural Colombia	5-point	SMN18	Cookies made from either 15% or 20% bean flour, 15% cassava, and 70 or 65% wheat flour	Acceptable	Cabal G et al (2014)^54^
Households in rural Rwanda, N = 1809	7-point	RWV3116 RWV3006 RWV2245 MAC44 Local variety (control)	Ground Whole beans	Acceptable	Muange & Oparind, (2018); Murekezi et al (2017); Oparinde, Birol et al (2016); Oparinde et al (2015, 2017, 2018)^55–60^
Panelists in Uganda, N = 50	9-point	ROBA1 K131 (control)	Porridge and sauce from pure or composite extruded or malted/roasted flour	Acceptable	Nkundabombi et al (2016)^61^
Adult consumers and bean sellers, rural Guatemala, N = 360	7-point	Super chiva ICTA-Unapu (control)	Raw or cooked	Acceptable	Perez et al. (2015, 2017, 2018)^62–64^
Children (N = 75) and adults (N = 173), urban and rural Colombia	4-point. Facial (children) 5-point, facial (adults)	SMR 4, SMN 18, SMC 14, and SMB 17 Testigo (control)	Cooked with rice	Acceptable among children but not adults	Tofino et al (2011)^52^

a Foods were considered acceptable if they had an overall sensory acceptability score of ≥70%.

b Control refers to a nonbiofortified, non-industrially fortified, conventional crop.

**Table 7:** Summary of studies investigating sensory acceptability of provitamin A-cassava via hedonic score testing

Population, N	Hedonic scale	Varieties investigated	Food product	Acceptability result^a^ for biofortified food products	Reference
Adult men in rural Nigeria, N = 20	9-point	TMS 1358	Gari paste Eba fermented 1 d–4 d	Acceptable	Abiodun et al (2020)^67^
Adults in rural Nigeria, N = 50	9-point	High-quality yellow cassava starch	Gruel with 60%–100% cassava starch and 0%–40% partially defatted soybean flour	Acceptable	Alake et al (2016)^68^
Staff and graduate students at university in Nigeria, N = 12	9-point	NR	Ogi gruel with 88%–94% cassava root starch and 0.3%–12% whole egg	Acceptable	Awoyale et al (2016)^69^
Semi-trained panelists in rural Nigeria, N = 12	9-point	TMS 01/1368 TMS 419 (control^b^)	Bread (20% cassava, 80% wheat)	Acceptable	Awoyale et al (2019) (pre-print)^70^
Adults (18-83 years) in suburban Nigeria	9-point	TMS 01/1368 TMS 01/1371 TME 419 (control)	Eba with or without red palm oil or Fufu	Acceptable	Bechoff et al (2018)^71^
Adult consumers in Nigeria, N = 30	9-point	TMS 07/0593 White conventional (control)	Pasta with or without amaranth vegetables	Acceptable without amaranth vegetables	Lawal et al (2021)^72^
Adults in Brazil, N = 134	9-point	BRS Dourada BRS Gema de ovo BRS Jari Hybrid 2003 1411	Dehydrated chips, ± onion and parsley flavoring	Acceptable: BRS Jari and Hybrid 2003 1411 with onion and parsley flavor and plain Hybrid 2003 1411	Oliveira et al (2017)^66^
Adults in rural Nigeria, N = 671	5-point	Light yellow Very deep yellow Local variety (control)	Gari Eba	Acceptable	Oparinde, Banerji, et al. (2016)^65^

a Foods were considered acceptable if they had an overall sensory acceptability score of ≥70%.

b Control refers to a nonbiofortified, non-industrially fortified, conventional crop. *Abbreviations*: NR, not reported.

**Table 8:** Summary of studies investigating sensory acceptability of iron- ± zinc-biofortified pearl millet via hedonic score testing

Population, N	Hedonic scale	Varieties investigated	Food product	Acceptability result^a^ for biofortified food products	Reference
Children 10 y–14 y) in peri-urban India, N = 400	5-point	Dhanashakti/ICTP-8203Fe	Khichdi	Acceptable	Anitha et al (2019)^74^
Adults in rural India, N = 452	5-point	Dhanashakti/ICTP-8203Fe Local variety (control^b^)	Grain Bhakri	Acceptable	Banerji et al (2015, 2016)^44^^,^^75^
Breastfeeding mothers in rural India, N = 52	9-point	Dhanashakti/ICTP-8203Fe DG9444 (control)	Idli Mudde Porridge	Acceptable	Gannon et al (2019)^24^
Adults in rural Burkina Faso, N = 24	5-point	Tabi GB 8735 Local Gampela (control)	Tô (whole or decorticated millet) Pancakes (whole or decorticated millet) Gruel (whole or decorticated millet)	Acceptable: whole millet pancakes made with GB 8735; whole millet gruel; decorticated millet pancakes	Hama-Ba et al (2019)^76^
Mothers in urban slums of India, N = 38	9-point	Dhanashakti/ICTP-8203Fe 9444 (control)	Cookies Peanut laddu Sheera Churma laddu Cake Nankhatai Porridge Puranpoli Khichdi Upma Dhokla Idli Vegetable cutlet Kothimbir wadi Pav Bhaji Pakoda Vada	Acceptable	Huey et al (2017)^14^^,^^15^
Semi-trained panelists in India, N = 10	9-point	AHB (Aurangabad Hybrid Bajra) MRB (Maharashtra Rabi Bajra) (control)	Cookies (whole wheat flour: raw pearl millet flour at 60:40 or 70:30 ratios)	Acceptable	Kale et al (2018)^73^

a Foods were considered acceptable if they had an overall sensory acceptability score of ≥70%.

b Control refers to a nonbiofortified, non-industrially fortified, conventional crop.

**Table 9:** Summary of studies investigating sensory acceptability of zinc-biofortified rice via hedonic score testing

Population, N	Hedonic scale	Varieties investigated	Food product	Acceptability result^a^ for biofortified food product	Reference
Adults in urban Bolivia (N = 97) or Colombia (N = 146)	7-point	PCT-25-C2-329-4-2SR-5P CT22154-9P-1SR-1P-3SR Local variety MAC-18 (control^b^) CICA4 (control)	Cooked	Acceptable	Woods et al (2020)^79^
Adult panelists in India, N = 10	5-point	Karidaddi Makam IVT (SHW) 91 Badshahbhog BI 43 BI 33 (control)	Cooked	NR^c^	Yareshimi et al (2013)^78^

a Foods were considered acceptable if they had an overall sensory acceptability score of ≥70%.

b Control refers to a nonbiofortified, non-fortified, conventional crop.

c In the study Yareshimi 2013, mean sensory scores of cooked biofortified rice varieties are given in Figures 1 and 2, but it is not clear how these means were calculated. It appears that a “Figure 3” is missing from the paper, which may contain each parameter's individual score. We have included the mean score here for reference. *Abbreviations:* NR, not reported.

**Table 10:** Summary of studies investigating sensory acceptability of iron-biofortified cowpeas, iron-biofortified lentils, zinc-biofortified wheat, and composite meals made from combinations of multiple provitamin A-, iron-, and zinc-biofortified crops, via hedonic score testing

Cowpeas					
Population, N	Hedonic scale	Varieties investigated	Food product	Acceptability result^a^ for biofortified food product	Reference
Untrained adults in urban Brazil, N = 100	9-point	BRS Xiquexique	Cheesebread with 5.6%–8% cowpea flour	Acceptable	Cavalcante et al (2016)^80^
**Lentils**					
Breastfeeding mothers in rural India, N = 52	9-point	Pusa Vaibhav Moitree (control^b^)	Sambar	Acceptable	Gannon et al (2019)^24^
**Wheat**					
Breastfeeding mothers in rural India, N = 52	9-point	BHU-6 HD2967 (control)	Porridge	Acceptable	Gannon et al (2019)^24^
**Combinations of multiple crops (iron- and zinc-biofortified pearl millet, provitamin A-biofortified OSP; iron-biofortified lentils, zinc-biofortified wheat)**					
Breastfeeding mothers in rural India, N = 52	9-point	Dhanashakti (pearl millet)Kamalasundari (OSP) BHU-6 (wheat) Pusa Vaibhav (lentils) DG9444 (control pearl millet) HD2967 (control wheat) Moitree (control lentil) White sweet potato (control)	Pulao Kesari Poli	Acceptable	Gannon et al (2019)^24^

a Foods were considered acceptable if they had an overall sensory acceptability score of ≥70%.

b Control refers to a nonbiofortified, non-industrially fortified, conventional crop.

### Summary of studies using hedonic scales to assess acceptability

#### Provitamin A–biofortified orange sweet potato

Provitamin A–biofortified OSP was most represented by studies (Table 4^17^^,^^19–33^, see Table S1 in the Supporting Information online), with 15 studies reporting on its sensory acceptability. Countries discussed included Rwanda, Uganda, India, South Africa, Malawi, Brazil, Nigeria, Kenya, and Tanzania. The most common population surveyed was adults from rural areas, although breastfeeding mothers, children, and adults from urban populations were also included. Beauregard was the most commonly examined variety, though some studies did not report the exact variety tested. Other OSP varieties included Kamalasundurai, A45, Chipika, Kadyaubwerere, Zondeni, Resisto, Ejumula, SPK004/1/1, S-61, S-594, S-1156, S-1281, SV-98, 362–7, IGSP-15, CIPSWA-2, 187017–1, 440038, 440127, 420027, ST-14, 90/101, and Karote DSM. Controls, if used, included nonbiofortified varieties of lighter-colored sweet potato (such as white sweet potato [WSP], including Tanzania variety, Nakakande WSP, local WSP varieties, Kenya variety, Polista, and Sinia B cream-fleshed sweet potato A40), and seed (nonsweet) potato (Dutch Robyjin). Food products included boiled (with and without skin), chutney, rasam, thick shake, barfi, payasam, sharbat, dry enriched cookie, chips, doughnuts, veggies, bread, raw, fried, and cake.

##### Biofortified OSP vs nonbiofortified OSP

Chutney, rasam, thick shake, barfi, payasam, and sharbat made from either Kamalasundurai or WSP were acceptable among breastfeeding mothers in rural India.^24^ Several studies across Africa found boiled OSP (A45, Chipika, Kadyaubwerere, Zondeni, Karote DSM, Resisto) or the control (A40, Kenya, Polista, Sinia B) were acceptable.^19^^,^^25^^,^^27^ Chips or crisps made with Kemb 10 or Dutch-Robyjin – a regular potato with similar moisture content to that of sweet potato – using either corn oil or palm oil were all acceptable.^26^ One study examined acceptability of boiled sweet potato using a 9-point hedonic scale; however, quantified results were not reported.^20^^,^^21^

##### Biofortified OSP: variety or preparation methods comparison

In consuming boiled OSP, particular varieties (187017–1, 420027, and an unreported OSP variety) were less acceptable than other varieties (see Table S1 in the Supporting Information online).^27^^,^^30^ Boiling without skin for 15 minutes was less acceptable than 10 minutes, or boiling for either 10 or 15 minutes with skin.^32^ Frying OSP was acceptable.^32^ Juiced OSP alone was more acceptable than juiced OSP with pineapple juice added.^23^ An unreported OSP variety was roasted or prepared as tamales, pesada, or soda; all were acceptable.^23^ Baked goods made with varying amounts of Beauregard flour, including cake (36 g to 64 g flour/100 g plus oil [1.3 g/100 g–12.7 g/100 g]),^34^ bread (20%–60% flour),^31^ and cookies (extruded or non-extruded flour) were all acceptable.^28^

##### Biofortified OSP: other sensory acceptability methods

In western Kenya, n = 501 caregivers of children under 5 years of age or pregnant women used a 5-item JAR scale (including anchors of “much too little,” “too little,” “just about right”, “too much”, and “much too much”) to rate acceptability of the VITAA varietal of OSP, prepared by boiling.^35^^,^^36^ Several sensory attributes (including sweetness, smell, color, texture/softness, taste, and crumbliness) were compared between VITAA and nonbiofortified white or yellow sweet potato. Generally, the majority of participants rated each OSP attribute “just about right”, with the exception of an even split of 68% of participants rating “smell” as being “too little” or “just about right” (34% each).

One study in a population of 15 trained panelists in Brazil used the QDA measure to assess the sensory acceptability of OSP (variety: Beauregard) processed into chips, then stored in various conditions.^37^ In brief, storage with nitrogen, in either polyester/aluminum foil/low-density polyethylene (PET/Al/LDPE), biaxially oriented polypropylene (BOPP)/metallized (met) BOPP without an oxygen scavenger, and the latter with an oxygen scavenger showed less significant sensory alterations (in flavor, odor, color, crispness) during 207 days of storage, than chips packed without nitrogen in BOPP/metBOPP after 153 days or chips packaged with nitrogen in PETmet/LDPE for 184 days. The QDA method entailed using a 9-cm nonstructured scale ranging from 0 to 9, with a score of ≥4.5 defined as product rejection (therefore, scores closer to 0 were considered better sensory acceptance). The extremes of the scales for each attribute were: color (0 = intense orange, 9 = light yellow); odor (0 = characteristic, 9 = odd); oxidation odor (0 = absent, 9 = strong); flavor (0 = characteristic, 9 = not characteristic); oxidation flavor (0 = absent, 9 = strong); crispness (0 = crunchy, 9 = limp); overall quality (0 = excellent, 9 = dreadful). This study was able to determine the best storage conditions for OSP chips to minimize oxidation and optimize palatability.

#### Pro-vitamin A–biofortified maize

Thirteen total studies reported on the sensory acceptability of provitamin A–biofortified maize (Table 5^25^^,^^38–51^, see Table S2 in the Supporting Information online). All of the studies covered populations in Africa, with 7 studies specifically targeting South Africa. Other regions included Nigeria, Saharan Africa, Ghana, Zambia, and Mozambique. Age groups surveyed included nursing mothers, adolescents, adults, and secondary school children. Only 1 study reported including trained individuals.^40^ Of the studies that reported the level of development in the area surveyed, all were in rural areas. Three studies did not report the exact biofortified variety used, but those reported included A0905-32, PVAH-62, PVAH-27–49, PVAH-1–26, PVAH-50–75, PVAH-79–100, PVA A, KP-78, KP-79, and KP-77. Controls, if used, included nonbiofortified varieties of white maize (such as WE-3172, SC-701, or unreported local varieties) or common yellow maize. Food products included porridge (stiff and thin), boiled whole cassava root, phutu, amahewu, kenkey, maize snack, samp, and nshima.

##### Biofortified maize vs nonbiofortified maize

The fermented beverage, Amahewu, was not well accepted when made with either biofortified or control maize,^42^ but adding roasted Bambara flour resulted in acceptability in both biofortified and nonbiofortified forms.^43^ Phutu, a crumbly maize porridge, was acceptable when made from either PVA pool A, PVA pool H, or the control white maize,^41^^,^^51^ but was not acceptable when prepared from KP-78, KP-79, or KP-77.^51^ Serving PVA A– or control WE-3172–based phutu alongside chicken curry or cabbage curry was acceptable, but PVA A–based phutu served with bambara groundnut curry was not acceptable, while control WE-3172–based phutu served with the same curry was acceptable.^25^ Kenkey dough made from biofortified orange or yellow maize, as well as white control maize, was acceptable.^44–46^ Nshima porridge made from biofortified orange maize was less acceptable than nshima porridge made from an also non-acceptable isogenic white maize, or an acceptable local white maize,^38^ but nshima made from prototype high provitamin A orange maize, or 2 controls, yellow and white maize, were all acceptable.^50^ Thin porridge and samp (coarse corn meal) made from KP-78, KP-79, or KP-77 was less accepted than the same dishes made with the white maize control SC-701.^51^ Finally, porridges made from various proportions of fermented A9895032 maize flour or common yellow maize were all acceptable.^39^

##### Biofortified maize: variety or preparation methods comparison

Maturation time of the maize appeared to influence the acceptability of 8 boiled maize varieties (see Table S2 in the Supporting Information online), with younger maize (harvested 20 days after pollination) being accepted across all 8 varieties.^40^ Only 2 out of the 8 varieties were still acceptable at 27 days pollination, while only 1 was acceptable at 34 days after pollination. Maize snacks made with 5 varieties of extruded maize with or without amaranth leaf powder scored consistently low for sensory acceptability.^48^

##### Biofortified maize: other sensory acceptability methods

Finally, 1 study that reported hedonic scale results among secondary school children (n = 54) and adults (n = 50) also used a paired preference test among these groups as well as among preschool (n = 52) and primary school children (n = 56)^51^ to measure sensory acceptance of biofortified maize made into phutu, thin porridge, and samp in KwaZulu-Natal. Among preschool and primary school children, the biofortified maize variety KP-79 and the control, a commercial white maize (SC-701), were tested. Preschool children preferred the biofortified versions of all 3 foods. Conversely, primary school children had no preference for phutu and samp, but preferred thin porridge made with control maize. In testing the additional biofortified varieties, KP-78 and KP-77, secondary school children and adults strongly preferred control white maize relative to the biofortified yellow varieties.

#### Iron-biofortified beans

Six studies reported on the sensory acceptability of iron-biofortified beans (Table 6^52–64^, see Table S3 in the Supporting Information online). Three studies covered populations in Colombia, and 1 study covered populations in each of Rwanda, Uganda, and Guatemala. Among studies reporting the level of development in the area surveyed, both rural and urban areas were included. A greater percentage of the studies on biofortified beans, as compared with studies on other biofortified crops, surveyed children. Other populations included households, panelists, adult consumers, and bean sellers. A diverse range of varieties were studied, including BIO-101, BIO-107, SMN18, RWV3116, RWV3006, RWV2245, MAC44, ROBA1, Super chiva, SMR4, SMC14, and SMB17. Control, nonbiofortified varieties included local unreported types, Diacol-Calima, K131, Mutiki, ICTA-Unapu, and Testigo. Food products included plain cooked beans, beans cooked with vegetable sauce, beans baked into cookies, beans made into porridge, and beans ground into grain.

##### Biofortified beans vs nonbiofortified beans

Bean varieties BIO-101 and BIO-107 or a local control, cooked and served with vegetable sauce, were acceptable.^53^ The ROBA1 or control K131 varieties, made into porridge or ground and made into a bean sauce using different flour processing methods were acceptable.^61^ The MAC 44 and RWR 2245 varieties were cooked (without additional details) and compared with the local control Mutiki, across several testing populations, including rural households and urban consumers, and all products were found to be acceptable.^62–64^ Another study examined Super chiva variety beans and control, ICTA-Unapu; both were acceptable as cooked beans (no other preparation details reported), with or without nutrition information given.^55–60^ Finally, rice with cooked beans, including SMR 4, SMN 18, SMC 14, or SMB 17, or control Testigo, were acceptable among children, but not among adults.^52^

##### Biofortified beans: variety or preparation methods comparison

Variety SMN 18 made into cookies with either 15% or 20% bean flour was acceptable.^54^

#### Pro-vitamin A–biofortified cassava

A total of 8 studies reported on the sensory acceptability of provitamin A–biofortified cassava (Table 7,^65–72^ see Table S4 in the Supporting Information online). Seven studies were conducted on populations in Nigeria, and 1 study was conducted on a population in Brazil. All studies were undertaken in rural settings. All the studies had populations consisting of adults, including university staff, graduate students, and semi-trained panelists. Additionally, all studies except for 1^65^ used the 9-point hedonic scale. Some studies did not report the exact variety of cassava used, but those reported included TMS 1358, TMS 01/1368, TMS 07/0593, TMS 01/1371, BRS Dourada, BRS Gema de ovo, BRS Jari, and Hybrid 2003 1411, as well as a custard paste made from High Quality Yellow Cassava Starch (HQYCS). Control, nonbiofortified white cassava varieties included TME-419, or undisclosed local varieties. Food products included gari paste, eba, ogi gruel, bread, pasta, dehydrated chips, and gari.

##### Biofortified cassava vs nonbiofortified cassava

From a non-peer-reviewed pre-print, bread made with 20% of either TMS 01/1368 cassava or control TME 419, and 80% wheat were accepted.^70^ Pasta made from TMS 07/0593 was more accepted than pasta made from control white cassava.^72^ Eba made from TMS 01/1368 or TMS 01/1371, or undisclosed very deep yellow or light yellow cassava, were all acceptable, as was eba made from control white cassava.^65^^,^^71^ Gari made from very deep yellow or light yellow cassava, and fufu made from TMS 01/1368 or TMS 01/1371, and their control cassava counterparts, were all acceptable.^65^^,^^71^ Fufu was also acceptable, made from either TMS 01/1368, TMS 01/1371, or control TME 419.^71^

##### Biofortified cassava: variety or preparation methods comparison

Chips made from BRS Dourada, BRS Gema e ovo, BRS Jari, or Hybrid 2003 1411 cassava with the addition of onion and parsley flavoring showed greater acceptability than similar chips without the flavoring.^66^ Eba and gari paste made from TMS 1358 were acceptable when fermented for 0 days–4 days; as a note, at 4 days, hedonic scale parameters moldability and overall were rated as not acceptable.^67^ Gruels with varying proportions (60%–100%) cassava starch, and either 0%–40% partially defatted soybean flour^68^ or 2%–12% whole egg,^69^ were also all acceptable.

##### Biofortified cassava: other methods for determining sensory acceptability

Two studies in this review incorporated the JAR sensory analysis.^35^^,^^36^^,^^71^ In Nigeria, West African food products, ie, eba and fufu, were made from either biofortified crops or the control (ie, the nonbiofortified conventional crop or industrially fortified conventional crop–based products and/or other industrially fortified products eg, fortified oil) cassava and tested for sensory acceptability using a 3-level JAR scale among n = 122 adult consumers.^71^ The attributes included color (being too white, JAR, or too yellow); fermented odor (too weak, JAR, too strong); texture (too soft, JAR, too hard). Biofortified varietals included TMS 01/1368 and TMS 01/1371, while the control conventional cassava was TME 419 processed with or without red palm oil to fortify it with vitamin A. In general, consumers were satisfied with the color, smell, and odor of the biofortified cassava-based eba and fufu, and the authors noted that the addition of red palm oil in the fortified foods may have caused an untoward odor and softened the texture, leading to greater liking for biofortified and nonfortified cassava food products.

#### Iron-biofortified pearl millet

In total, 6 studies reported on the sensory acceptability of biofortified pearl millet (Table 8,^14^^,^^24^^,^^44^^,^^73–76^ see Table S5 in the Supporting Information online). Five studies were undertaken on populations in India, and 1 study was undertaken on a population in Burkina Faso. The population groups surveyed included adults, breastfeeding mothers, nonbreastfeeding mothers, and semi-trained panelists. Among the studies that reported the level of development in the area surveyed, all were rural except 1.^14^ The most common biofortified variety tested was Dhanashakti (ICTP-8203Fe), but Tabi, GB 8735, and Aurangabad Hybrid Bajra were also represented. Controls included undisclosed local varieties, DG94444, local Gampela, and MRB (Maharashtra Rabi Bajra). Food products included grain, bhakri, idli, mudde (ball), porridge, tô, pancakes, gruel, cookies, peanut laddu, sheera, churma laddu, cake, nankhatai, puranpoli, khichdi, upma, dhokla, idli, vegetable cutlet, kothimbir wadi, pav, bhaji, pakoda, and vada.

##### Biofortified pearl millet vs nonbiofortified pearl millet

Tô and pancakes made with decorticated or whole pearl millet varied in acceptability: tô was only accepted made with control (local Gampela) pearl millet, while pancakes were consistently acceptable when made with variety GB 8735, either whole or decorticated, but only acceptable when variety Tabi was decorticated.^76^ Bhakri made from Dhanashakti or a local variety^75^^,^^77^; idli, mudde, and porridge,^24^ and several dessert items (cookies, laddu, sheera, cake, nankhatai, sweet porridge, and puranpoli) and savory items (khichdi, upma, dhokla, idli, vegetable cutlet, kothimbir wadi, thempla, pav bhaji, pakoda, and vada),^14^^,^^15^ made from Dhanashakti or control DG 9444 varieties were all acceptable. Finally, cookies made from either AHB or control MRB pearl millet were acceptable, either in a 70:30 or 60:40 ratio of whole wheat flour and germinated pearl millet flour.^73^

All other biofortified pearl millet food products were acceptable.

##### Biofortified pearl millet: variety or preparation methods comparison

Khichdi made from Dhanashakti was found to be acceptable.^74^

##### Biofortified pearl millet: other sensory acceptability methods

Two studies measured young (6-month-old–24-month-old) children’s acceptance of biofortified pearl millet through biofortified pearl millet intake amount in rural Southern India^24^ and in the urban slums of Mumbai^14^^,^^15^ (see Table 8 for varieties). Children were given a weighed sample (additional servings were available ad libitum) and were fed until the child refused food. The remaining food was weighed to calculate the net amount consumed, with a greater quantity of food consumed correlating to a higher acceptability of that food. In general, there were no differences in the amount of consumption of foods prepared with the biofortified crops or with control crops across both studies, indicating the acceptability of food products made with biofortified pearl millet among children.

#### Zinc-biofortified rice

In total, 2 studies reported on the sensory acceptability of biofortified rice (Table 9,^78^^,^^79^ see Table S6 in the Supporting Information online). The countries surveyed included Bolivia, Columbia, and India. The biofortified varieties included PCT-25-C2-329–4-2SR-5P, CT22154-9P-1SR-1P-3SR, BF14AR021, BF14AR035, Karidaddi, Makam, IVT (SHW) 91, Badshahbhog, and BI 43; the control nonbiofortified varieties included local varieties MAC-18, CICAA4, and BI 33. The only age group represented was adults, and the only processing method included was cooked, which was found to be acceptable in 1 study,^79^ while acceptability results were not fully reported in the other study.^78^

#### Iron cowpeas, iron lentils, zinc wheat, and combinations of multiple crops

Cowpeas,^80^ lentils,^24^ and wheat^24^ were investigated in 1 study each (Table 10,^24^^,^^80^ see Tables S7–S10 in the Supporting Information online). The countries surveyed included Brazil (cowpeas) and India (lentils, wheat). The age groups represented were adults (in Brazil) and breastfeeding infants and their mothers (in India). The lentil and wheat data came from the same study. Biofortified lentils (biofortified: Pusa Vaibhav; control: Moitree) made into sambar were acceptable, as was biofortified wheat (biofortified: BHU-6; control: HD2967) made into porridge.^24^ However, the study examining cowpeas (variety BRS Xiquexique) did not include a comparison with a control nonbiofortified crop in the methodology; instead, cheese bread made with either 5.6% or 8.0% cowpea flour were both found to be acceptable. One study compared multiple biofortified crops–based dishes with the same dishes made with nonbiofortified crops (see Table S10 in the Supporting Information online), including pulao, kesari, and poli; all were rated acceptable.^24^

## DISCUSSION

### Main findings

This systematic review summarized the results of testing sensory acceptability directly in target populations across 10 conventionally biofortified food products, including those made from OSP, maize, beans, cassava, pearl millet, rice, cowpeas, lentils, wheat, and combinations of multiple biofortified crops into a single composite meal. From the current evidence base, the findings suggest that foods and food products made from biofortified crops generally had acceptable sensory characteristics, reaching hedonic score-based acceptance indices of at least 70% and showing sensory acceptability using other methods, across children, adolescents, and adults.

The general acceptance of most biofortified foods among consumers in this review is consistent with findings on the acceptance of biofortified OSP, maize, cassava, pearl millet, beans, and rice in previous reviews on biofortified crop acceptance in lower-to-middle income countries,^3^^,^^4^ but these reviews differed in that they did not examine lentils, wheat, cowpeas, or composite meals, likely due to lack of available data. Furthermore, these reviews differed in that they included all types of biofortified crop (ie, those biofortified by not only conventional breeding, but also agronomic methods and genetic engineering); included the result of hypothetical acceptance from customers’ own perceptions of the crop but not direct experience with the crop; and did not distinguish the biofortified varieties tested.

### Reasons for non-acceptance of certain biofortified foods and recommendations

Only a few studies found that certain biofortified foods had an overall “not acceptable” rating, which may aid further development of that food product, such as changing the additional ingredients that are added to the food product. For example, in testing OSP juices, adding pineapple juice for sweetness resulted in lower acceptability than adding plain OSP juice; for maize amahewu, adding wheat bran or malted maize inoculum was not acceptable, but amahewu made with roasted Bambara flour was acceptable, suggesting multiple formulations of a given food product may need to be tested to determine acceptability. Chips made from cassava showed improved acceptability with the addition of onion and parsley flavoring, which may be a factor for manufacturers to consider in designing processed foods.

Other methods for aiding development of the food product may involve adjusting the methods of growing, preparation, or storage. For example, OSP boiled with or without skin for 10 minutes or with skin for 15 minutes was acceptable, but OSP boiled without skin for 15 minutes was not acceptable. Maize that was allowed to pollinate for 20 days and boiled was more acceptable than maize that was allowed to pollinate for 27 days–34 days and boiled. For pearl millet, decorticated grains were more accepted than whole-grain versions for some food products (gruel); other foods, such as pancakes, were found to be unacceptable using either decorticated or whole grains. For storing OSP chips, particular packaging is needed to maintain optimal sensory characteristics and acceptability after storage for several months and to minimize undesirable flavors and odors due to oxidation. These findings may also aid developers in finding the ideal growing, preparation, and storage techniques.

Particular varieties of OSP, maize, and beans were more acceptable than others, indicating that producers and processors should select only varieties shown to be more acceptable by consumers. For OSP and maize, this was apparent when the crop was boiled whole, while for beans this was shown only when beans were made into porridge or sauce. Conversely, the studies included did not indicate variety-informed sensory acceptability for whole boiled cassava. In preparing boiled OSP, 2 out of 15 varieties tested were not acceptable, suggesting that variety may be an important consideration for developing food products for particular processing techniques.

Bias against certain sensory attributes may also explain lower acceptance for certain foods. For example, 1 study noted that it is possible, from accompanying focus group discussions, that preconceptions about biofortified maize – for example, yellow maize being associated with animal feed – may bias older individuals towards preferring white maize.^51^ This study showed that biofortified maize may be acceptable in preschool programs but would require additional strategies (eg, intensive nutrition education programs) to incorporate into the diets of older individuals.

### Gaps and limitations

In our review, several crops, including rice, cowpeas, wheat, and lentils, were represented by only 1 to 2 studies each, indicating a gap in the literature, possibly informed by limited dissemination. However, investigating the overall acceptability of mineral-biofortified crops from only a few studies limits the overall generalizability; diverse populations and settings likely have variability in sensory acceptability. Other gaps include the populations tested (few studies included preschool children, schoolchildren, or adolescents) and testing the acceptability of composite meals including multiple kinds of biofortified crops, which was done in only 1 study. For example, rice and beans is a commonly consumed meal in Latin America, which is an area where biofortified crops have been introduced^81^; therefore, this meal could be made using zinc-biofortified rice and iron-biofortified beans and tested for sensory acceptability. Finally, only 1 study appeared to examine the sensory acceptability of a food product that was stored for an extended time period – a major gap given that crops are not always going to be consumed just after harvesting and cooking or otherwise processing, considering seasonality.

While this review found data for 10 types of biofortified crops, the sensory acceptability of other crops currently being developed by HarvestPlus was not found in the evidence base developed from our literature search – for example, vitamin A–biofortified banana and plantains; iron-biofortified irish potato; zinc-biofortified sorghum.^81^ These are in dissemination, national performance trials for release, or being tested, or released across several countries (depending on crop)^81^; it will be important to generate data on the sensory acceptability of these crops as an early step to incorporating such crops.

## CONCLUSIONS

This review adds detailed data on sensory acceptance of biofortified food products and recommends potential paths for processors of biofortified crops to help improve acceptability. Generally, biofortified crops were well accepted. Lack of acceptance could be attributed to specific ingredients added to the food product, to particular preparation methods, or to biased perceptions regarding the food. Several research gaps remain, including sensory acceptability studies in diverse populations and settings on: biofortified rice, cowpeas, lentils, and wheat; combinations of multiple biofortified crops into composite meals; stored biofortified food products; and foods made from newer biofortified crops currently being introduced across the world. Studies evaluating new biofortified crops, crop varieties, and food products should include a formal sensory acceptability evaluation where feasible, to inform programmatic scale-up and implementation and ensure success across diverse populations and settings.

## Supplementary Material

nuad100_Supplementary_Data

## Supporting Information

The following Supporting Information is available through the online version of this article at the publisher’s website.

*Table S1*  **Sensory acceptability for biofortified orange sweet potato**

*Table S2*  **Sensory acceptability for biofortified maize**

*Table S3*  **Sensory acceptability for biofortified beans**

*Table S4*  **Sensory acceptability for biofortified cassava**

*Table S5*  **Sensory acceptability for biofortified pearl millet**

*Table S6*  **Sensory acceptability for biofortified rice**

*Table S7*  **Sensory acceptability for biofortified cowpeas**

*Table S8*  **Sensory acceptability for biofortified lentils**

*Table S9*  **Sensory acceptability for biofortified wheat**

*Table S10*  **Sensory acceptability for multiple biofortified crops made into a composite meal**

*Table S11*  **PRISMA Checklist**
