# Correction to: Sensory acceptability of biofortified foods and food products: a systematic review

**DOI:** 10.1093/nutrit/nuad173

**Published:** 2024-01-13

This is a correction to: Samantha L Huey, Arini Bhargava, Valerie M Friesen, Elsa M Konieczynski, Jesse T Krisher, Mduduzi N N Mbuya, Neel H Mehta, Eva Monterrosa, Annette M Nyangaresi, Saurabh Mehta, Sensory acceptability of biofortified foods and food products: a systematic review, Nutrition Reviews, 2023; https://doi.org/10.1093/nutrit/nuad100

In the originally published version of this manuscript, the files for Supplementary Data section were missing for Tables S1-S10 and the links lead erroneously only to Table S11. The lacking material for Tables S1-S10 is now supplied to the paper and the links emended in the article.

